# Infusion of an acidified ethanolic—dextrose solution enhances urinary ammonium excretion and increases acid resilience in non—mechanically ventilated acidotic rabbits

**DOI:** 10.3389/fphys.2022.860042

**Published:** 2022-10-12

**Authors:** Patrick A. Walsh

**Affiliations:** Department of Physiology, School of Medicine, RCSI Bahrain, Al Muharraq, Bahrain

**Keywords:** acetate, acidosis, ammonia, herbivore, hyperammonaemia, kidney, rabbit, renal

## Abstract

Hitherto, the rabbit has long been known to have a very poor tolerance to non—volatile acid. In this study, we tested the hypothesis that acid resilience in the acidotic rabbit can be increased by enhancing the plasma availability of a naturally occurring volatile fatty acid, namely acetate. To ascertain the relative merits of the respiratory and renal systems in contributing to that resilience, we conducted our studies in non—ventilated and mechanically ventilated acidotic animals. Using ethanol as a feeder of acetate, and to counteract the antidiuretic effects of surgical interventions, we induced acidosis in anaesthetised rabbits, by intravenously infusing an acidified ethanolic dextrose solution. We observed very potent respiratory regulation of arterial blood pH coupled with a notable renal response by way of a 25-fold increase in urinary ammonium excretion in the non—ventilated group. In contrast, arterial blood pH plummeted much more rapidly in the mechanically—ventilated animals, but the compensated renal response was enormous, in the form of an 85 -fold increase in urinary ammonium output. Despite this significant adaptive renal response, the non -mechanically ventilated group of rabbits showed the greater acid resilience. This was attributed to an acetate stimulated flux through a series of metabolic pathways, generating supplementary buffer in the form of bicarbonate and ammonia, complemented by a robust respiratory response.

## 1 Introduction

The rabbit is an obligate nose breather. It respires mostly *via* the activity of its diaphragm. It has a normal resting respiratory rate in the region of 30–60 breaths min^−1^ and a minute volume of 0.25 L min^−1^ Kg^−1^ (Nowland et al., 2015), which is approximately 3-fold higher than that found in humans. Because the rabbit’s respiratory rate can increase to at least 200 breaths min^−1^ (Schroeder and Smith, 2011), the capacity of this animal’s respiratory system to remove volatile acid is considerable, in comparison with man.

Although a monogastric herbivore, the rabbit is a known hindgut fermenter. The microbial fermentation of fodder in its hindgut produces large quantities of volatile fatty acids (VFA), most notably acetate (Blaxter, 1962). It is the most abundant VFA found in rabbit blood, where it exists in millimolar concentrations (Bergman, 1990). Acetate reaches the liver in the portal circulation, where along with other VFA’s, it provides a large proportion of the energy requirements of the rabbit. Renal tubules and skeletal muscle of the rabbit have also been shown to have a high capacity to metabolize acetate (Blaxter, 1962, Dgelay et al., 1999). In addition to its role as an energy provider, acetate also fulfils a very important acid-base homeostatic function in the tissues of the rabbit, by virtue of its metabolic conversion into equimolar amounts of bicarbonate. The metabolic use of VFA’s, coupled with the intake of an alkaline ash diet, eliminates the non-volatile acidic burden on the kidneys of this species. The excretion of nutrient alkali, along with the net secretion of bicarbonate by the β-intercalated cells in the cortical collecting ducts (CCD), gives normal rabbit urine its unique acid-base characteristics, which include a high titratable alkalinity, a pH in the zone of 8 ± 0.4, a milky appearance due to the precipitation of calcium salts at alkaline pH, and trace concentrations of ammonia (Richterich et al., 1958; Walsh and O'Donovan, 2019a).

The rabbit has been shown to have a very poor tolerance to systemic inorganic acid loading, in comparison to man, rodents, and canines (Richterich et al., 1958; Bank, 1961; Weiner et al., 2015). The susceptibility of the rabbit to non-volatile acid loads has been attributed to its renal incapacity to generate adequate amounts of ammonia buffer. The buffering of H^+^ by the ammonia buffer, in preventing the urinary pH falling to ≤ pH 4.5, facilitates the continued secretion of large quantities of excess H^+^ into the collecting duct tubular fluid, while concurrently enabling the transport of equimolar quantities of HCO_3_^−^ back into the blood plasma from the epithelial cells. In more recent times, scientists have observed that the *de novo* synthesis of ammonia buffer can be significantly, though very modestly, increased through the infusion of inorganic phosphate (Yu et al., 1976; Walsh and O'Donovan, 2019a, Walsh and O'Donovan, 2019b).

Several key biochemical events have been proposed to collectively account for the very low rate of *de novo* synthesis of ammonia by renal cortex cells in the acidotic rabbit. Firstly, the ability of rabbit kidney tubule cells to utilize glutamine as a substrate is relatively poor because of weak phosphate—dependent glutaminase activity (Richterich and Goldstein, 1958; Chauvin et al., 1997). Secondly, a low maximum velocity of the renal mitochondrial dicarboxylate carrier within renal cells decreases the exit of malate from mitochondria, and consequently, decreases the upstream relief on the α-ketoglutarate dehydrogenase and glutamate dehydrogenase enzymes, resulting in a decrease in the rate of NH_4_^+^ secretion into the tubular fluid (Cheema-Dhadli and Halperin, 1979; Halperin and Cheema-Dhadli, 1980). Finally, the high glutamine synthetase activity, found in rabbit kidney nephrons (Chauvin et al., 1997), has been shown to trap ammonia as glutamine, thus rendering it unavailable for release into both the urine and the systemic circulation *via* the renal vein. Furthermore, glutamine synthetase activity in herbivorous kidneys has been shown to be increased following the administration of ammonium chloride (Michoudet et al., 1994).

Most acid-loading investigations in man and experimental animals have involved either the oral or intravenous administration of inorganic compounds such as ammonium chloride or hydrochloric acid. We became aware of a study by a research group (Leonard and Orloff, 1955), who had used an unusual acid - loading cocktail, containing 5% dextrose, 3% ethanol, and 0.1 M HCl, to induce metabolic acidosis in anaesthetized rats. We observed, however, that in rats infused with a solution containing 0.1 M HCl in 0.9% NaCl (unpublished studies), urinary ammonium excretion was substantially lower in comparison to the data reported in the paper by Leonard and Orloff (1955). Therefore, it became of great interest to us to determine if an acidified ethanolic dextrose solution, could have any significant impact on acid resilience, and urinary ammonium excretion, in an herbivorous species, such as the rabbit. Because the rabbit has a high resting minute volume, and the potential to expire large volumes of volatile acid under acidotic conditions, it was also decided to assess the renal response of acidotic rabbits, while they were under the control of mechanical ventilation.

## 2 Materials and methods

The ethical and animal husbandry procedures used in this study have been described in detail by us in a previous paper (Walsh and O'Donovan, 2020).

### 2.1 Ethical approval

The study was carried out at the Physiology Laboratories of the National University of Ireland Galway (NUIG) in Ireland. The animal experiments were performed in accordance with Directive 2010/63/EU. A license to undertake the animal experiments described in this paper, including ethical approval, was granted by the animal licensing committee at the Public Health Division of the Department of Health, Hawkins House, Dublin 2, Ireland.

### 2.2 Animal husbandry

Specific Pathogen Free (SPF) male New Zealand White (NZW) rabbits were used in the study. They were housed individually in cages, in an environmentally controlled room (20 ± 1°C; 35%–60% relative humidity; and 12:12 light-dark cycle). The animals were allowed free access to water and rabbit chow (Bio resources unit, Trinity College Dublin, Ireland).

## 3 Experimental overview

A total of 24 rabbits were used in the investigation. They were deprived of their solid food 12–15 h prior to the commencement of each experiment.

Following anaesthetization, each rabbit was placed in dorsal recumbency, on a small operating table, and secured in position using four limb restraining robes. Body temperature was maintained through-out the surgery at 39°C, using a homoeothermic blanket system with a flexible measuring rectal thermistor probe. A 12 FG de Pezzer urethral catheter was placed in the urinary bladder of each rabbit as described previously (Walsh and O'Donovan, 2016), to facilitate the collection of timed urinary specimens during each experiment. Intravenous infusions were made *via* an indwelling P.E. 50 cannula, which had been placed in the left jugular vein. The continuous infusion of solutions at a constant rate was facilitated through a Harvard syringe pump (model 2,681).

Arterial blood sampling was made through the left common carotid artery *via* an *in situ* P.E. 60 cannula, containing a luer lock. The cannula was filled with a heparinized saline solution (25 U ml^−l^ in 0.9%NaCl) between samplings. All blood samples were collected anaerobically from the cannulated artery in a 1 ml plastic disposable syringe whose dead space had been pre-filled with a sterile, neutral, isotonic heparin solution (5,000 I U ml^−1^).

A solution containing 5% dextrose and 3% ethanol was infused for 40 min, during which time baseline blood and urine samples were taken for analysis. Precisely at 40 min, a syringe valve to a solution containing 5% dextrose, 3% ethanol, and 0.1 M HCl was opened, and infusion was begun at a rate described in each experimental protocol. The 40 minute mark was taken as time zero, when computing the delivered dose of HCl to each animal. All animals were sacrificed by anaesthetic overdose, at the end of each experiment.

## 4 Experimental protocols

### 4.1 Four experimental protocols were evaluated in this study

#### 4.1.1 Protocol 1: Induction of metabolic acidosis in anaesthetized, non-ventilated animals, infused with 0.1 M HCl

A control group of nine rabbits (2.5–3.5 Kg) were pre-loaded with a volume of isotonic saline, equivalent to 3.5% of their respective body weights, over a 2 h period. This resulted in a mean urinary flow rate of 0.95 ± 0.08 ml min^−1^. The animals were then intravenously infused with 0.1 M HCl at a rate of 120 μmol min^−1^, until the blood bicarbonate approached 10 mmol L^−l^, as described previously (Yu et al., 1976; Walsh and O'Donovan, 2019a).

#### 4.1.2 Protocol 2: Induction of metabolic acidosis in anaesthetized, non-ventilated animals, infused with an acidified ethanolic-dextrose solution

Five rabbits (2.3–4.1 Kg) were pre-medicated with chlorpromazine hydrochloride (Largactil, Sanofi-Aventis: 2.5%, 25 mg Kg^−1^), followed 30–45 min later with intravenous pentobarbital (Nembutal, Abbot Laboratories: 5%, 15 mg Kg^−1^bw) through a marginal ear vein. Anaesthesia was maintained throughout the experiment by the intermittent injection of top-up doses of Nembutal. After the establishment of a normal rabbit arterial blood acid-base profile (Chapot et al., 1972), metabolic acidosis was induced in each animal by the infusion of a solution containing 5% dextrose, 3% ethanol, and 0.1 M HCl (469 mOsmoles Kg^−1^), at a rate of 87 μmol min^−1^.

#### 4.1.3 Protocol 3: Induction of acidosis in anaesthetized, mechanically ventilated animals, infused with an acidified ethanolic-dextrose solution

Four animals (2.8–3.9 Kg) were used in this study. To facilitate a more rapid induction of acidosis, the infusion rate was increased to 120 μmol min^−1^, and a mechanical respirator along with neuromuscular blockade were employed to control ventilation. The rabbits were infused with the same solution as described in Protocol 2.

The rabbits were placed under neuroleptic analgesia by the intramuscular injection of Hypnorm (0.5 ml kg^−1^bw). Additional top-up doses of Hypnorm were administered at intervals to maintain neuroleptic analgesia through-out the experiment.

On exposure of the trachea, a small incision was made, and a stainless-steel cannula was inserted into the trachea and secured in position with some surgical ligatures. The remaining two ports of the Y-shaped tracheal cannula were connected to a small animal benchtop intermittent positive pressure ventilator (IPPV). Following the administration of Pancuronium (0.1 mg Kg^−1^), through the marginal ear vein, mechanical ventilation was begun. The stroke volume of the IPPV was adjusted to the tidal volume, calculated for each rabbit according to its weight, using the formula of Guyton (Guyton, 1947). The stroke rate of the ventilator was adapted to the normal respiratory rate for a resting rabbit, ensuring that a normal rabbit arterial blood acid base status was established, prior to the commencement of any infusion. Respiratory muscle paralysis was maintained throughout the experiment by the periodic administration of small doses of Pancuronium.

#### 4.1.4 Protocol 4: Induction of acidosis in anaesthetized, mechanically ventilated animals, infused with an acidified ethanolic-dextrose solution, to which mannitol had been added as a diuretic

Six animals (3.1–4.4 Kg) were anaesthetized and artificially ventilated in a manner identical to the previous group. However, to achieve a more uniform urinary flow rate, mannitol was added to the infusion medium. Acidosis was induced by the intravenous infusion of a solution containing 5% dextrose, 3% ethanol, 5% mannitol, and 0.1 M HCl (755 mOsmoles.Kg^−1^), at a rate of 120 μmol min^−1^.

### 4.2 Measurement of blood acid-base parameters

Blood pH and pCO_2_ measurements were made using a Radiometer BMS-2 micro-electrode assembly with an acid-base analyzer. Bicarbonate concentrations were calculated using the Henderson-Hasselbach equation. Reference values for rabbit arterial blood pH and pCO2 at 39°C (Chapot et al., 1972), were used to establish a normal arterial blood acid-base status in the experimental rabbit groups, prior to the induction of acidosis.

### 4.3 Measurement of urinary ammonium, net acid excretion, and titratable acid excretion

Urinary specimens were assayed for ammonium as described previously (Walsh and O'Donovan, 2019a). Net acid excretion (NAE) and titratable acid (TA) excretion were determined as described by Cunarro and Weiner, 1974.

### 4.4 Measurement of the urinary excretion of aldosterone, K^+^, Na^+^

Urine specimens from artificially ventilated rabbits, who had been infused with a solution containing 5% dextrose, 3% ethanol, 5% mannitol & 0.1 M HCl, were assayed for K^+^ and Na^+^, using flame photometry, at the Clinical Biochemistry Division of University College Hospital, Galway, Ireland. Urine samples for aldosterone analysis were sent to Clinpath laboratories, in the United Kingdom.

### 4.5 Data analysis

Statistical analysis and presentation of the data was done using GraphPad Prism version 9.3.1 for Windows, GraphPad Software, La Jolla California United States, www.graphpad.com. The data are presented as means ± SD. Statistical comparisons were performed using a student’s paired *t* test, with statistical significance being taken as *p ≤* 0.05. Endnote—20.2.1 was used to compile the reference list.

## 5 Results

The arterial blood acid-base-gas (ABG) profile of each rabbit was closely monitored to ensure that, at the beginning of each investigation, the pre-infusion ABG values were as close as possible to the reference datapoints for this species (Chapot et al., 1972). These were: pH = 7.45 ± 0.02; pCO_2_ = 33.8 ± 1.8 mmHg; pO_2_ = 89 ± 5.0 mmHg, and [HCO_3_^−^] = 22.7 ± 1.2 mmol L^−1^, at 39°C. As the plasma volume in a 2.7 Kg rabbit computes to 95 ± 8 ml (Martínez-Ródenas et al., 1989), the actual plasma [HCO_3_^−^] in each rabbit, prior to the commencement of infusion, would have been circa 2.2 mmol.

To determine baseline blood and urinary acid—base data in acidotic rabbits, a control group of non-ventilated, isotonic saline—loaded, anaesthetised rabbits were rendered acidotic *via* the intravenous infusion of 0.1 M HCl over a 235—minute period (Protocol 1). During this time, arterial blood pH decreased to 7.24 ± 0.03 from a control reading of 7.44 ± 0.04 (*N* = 9), *p* < 0.01. A maximum urinary ammonium excretion rate of 0.05 ± 0.01 μmol min^−1^ (*N* = 9) was observed in this acidotic group of rabbits, which was very similar to data reported in a previous study of acidotic rabbits and guinea pigs (Richterich et al., 1958). A urinary net acid excretion of 0.95 μmol min^−1^ (*N* = 9), and a urinary pH of 5.1 ± 0.2 (*N* = 9) were recorded at the peak of the acidosis induction process.

**FIGURE 1:**
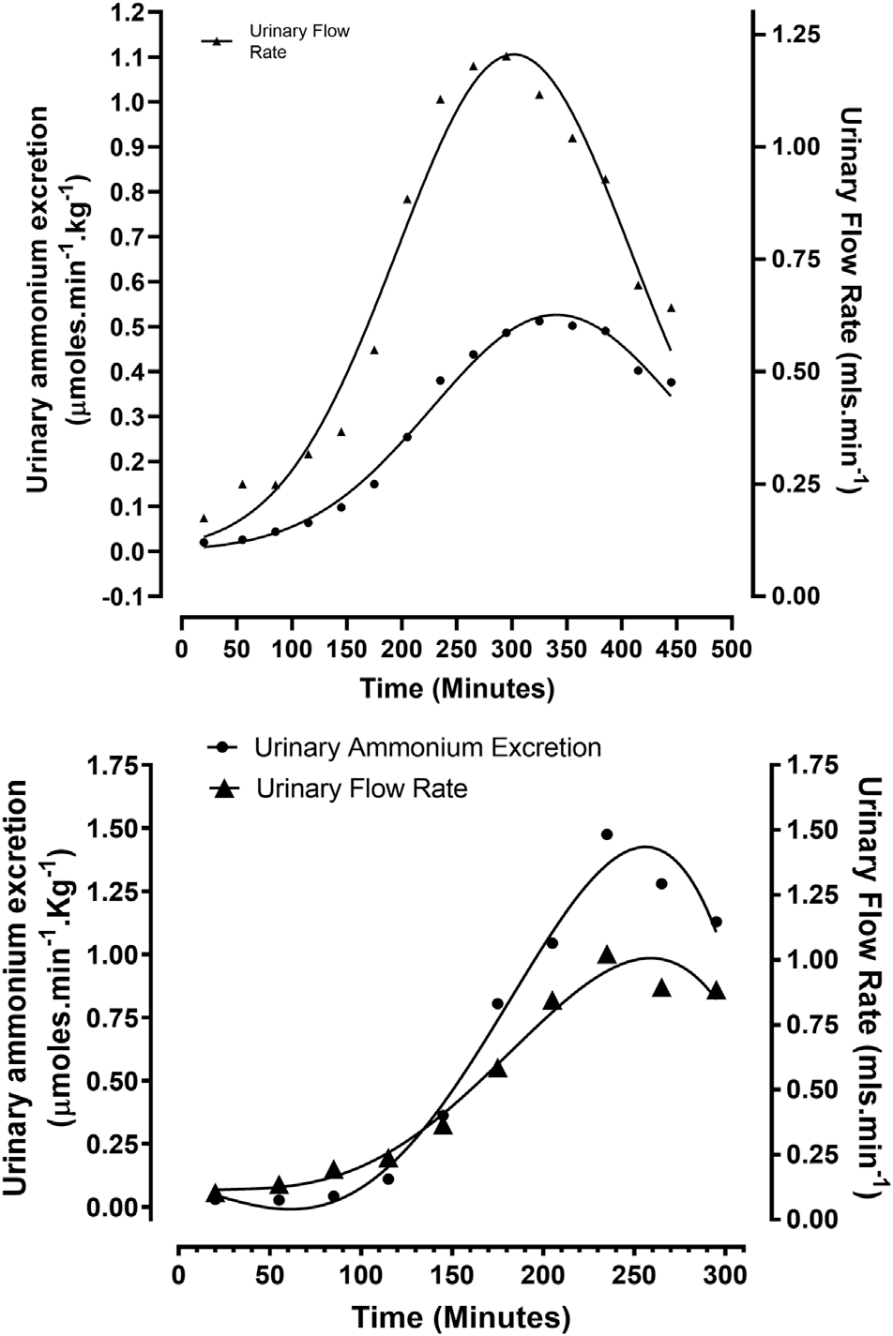
Correlation between urinary flow and ammonium excretion rates in rabbits treated according to Protocols 2 and 3: **(A)** Protocol 2 (N = 5): Ethanolic - dextrose + 0.1 M HCl, non – mechanically ventilated. **(B)** Protocol 3 (N = 4): Ethanolic - dextrose + 0.1 M HCl, mechanically ventilated.

A second group of non—ventilated animals, of similar age and weight to the previous group, were rendered acidotic *via* the infusion of a solution containing 5% dextrose, 3% ethanol, and 0.1M HCl, at a rate of 87 μmol min^−1^ (Protocol 2). The arterial blood pH decreased to 7.28 ± 0.07 from a control value of 7.47 ± 0.03 (*N* = 5) *p* < 0.01. Urinary flow rate increased from 0.07 ± 0.05 ml min^−1^ to reach a maximum steady output of approximately 1 ml min^−1^, between 235—355 min, after which it declined rapidly (Figure 1A). Urinary ammonium excretion increased to 0.50 ± 0.32 from 0.02 ± 0.01 μmol min^−1^. Kg^−1^ (*N* = 5), *p* < 0.01, representing a 25-fold increase in urinary ammonium output (Figure 1A). On a unit body weight basis, a 25-fold greater urinary ammonium output was observed in this group of acidotic rabbits (non-ventilated + acidified ethanolic-dextrose solution), in comparison with the control group of animals (non-ventilated + HCl).

A third group of four anaesthetised rabbits were mechanically ventilated with room air, while being rendered acidotic by the infusion of a solution containing 5% dextrose, 3% ethanol, and 0.1 M HCl at the rate of 120 μmol min^−1^ (Protocol 3). Artificial ventilation with room air facilitated a more rapid induction of metabolic acidosis by counteracting hyperventilation, induced by a rising pCO_2_ and falling arterial blood pH, due to intravenous acid loading. Arterial blood pH at 39°C decreased to 7.21 ± 0.07 from a reference value of 7.42 ± 0.03 (*N* = 4), *p* < 0.01. Urinary flow rate increased to 1.00 ± 0.50 from a control reading of 0.06 ± 0.05 ml min^−1^ (*N* = 4), *p* < 0.01. Urinary ammonium excretion rates increased almost 50-fold to 1.48 ± 0.84 from a baseline value of 0.03 ± 0.02 μmol min^−1^ Kg^−1^ (*N* = 4), *p* < 0.01 (Figure 1B). On a comparative unit body weight basis, the maximum urinary ammonium excretion observed in this group of rabbits (ventilated + acidified ethanolic − dextrose solution) was approximately 95—fold higher in comparison to the control group of animals (non-ventilated + 0.1 M HCl).

Because urinary ammonium excretion is known to be strongly flow—dependent in the rabbit (Richterich et al., 1958), mannitol was added as an osmotic diuretic to an acidified ethanolic dextrose solution. This cocktail was intravenously administered to a second group of anaesthetised, artificially ventilated rabbits at a rate of 120 μmol min^−1^ over a 235—minute period (Protocol 4). Urinary flow rate increased to 1.11 ± 0.16 at 235 min, from a control value of 0.28 ± 0.13 ml min^−1^ (*N* = 6), *p* < 0.01. Urinary ammonium excretion rates increased to 1.90 ± 1.22 from 0.08 ± 0.04 μmol min^−1^ Kg^−1^ (*N* = 6), *p* < 0.01 (Table 1), reflecting an approximately 125—fold higher urinary ammonium output relative to that shown by control group of animals (non-ventilated + 0.1 M HCl). Clearly, mannitol produced a more uniform urinary flow rate across the animals in this group. While urinary ammonium excretion increased significantly from baseline values for each of the two groups of rabbits over the course of the experimental period, no significant difference in urinary ammonium output was detected between those ventilated rabbits receiving acidified ethanolic—dextrose *versus* those being infused with acidified ethanolic − dextrose + mannitol (Table 1).

**TABLE 1:** Urinary ammonium excretion (µmoles.min^−1^ Kg.^−1^) in two groups of rabbits. Group 3 were infused with 5% dextrose, 3% ethanol, and 0.1 M HCl. Group 4 were infused with 5% dextrose, 3% ethanol, 5% mannitol and 0.1 M HCl. A multiple *t*-test comparison of the two groups showed no significan difference.

Time (minutes)	Group 3	Group 4	*p*-value
20	0.03 ± 0.02 (*N* = 4)	0.08 ± 0.04 (*N* = 6)	n.s
55	0.03 ± 0.02 (*N* = 4)	0.05 ± 0.03 (*N* = 6)	n.s
85	0.04 ± 0.04 (*N* = 4)	0.09 ± 0.07 (*N* = 6)	n.s
115	0.11 ± 0.09 (*N* = 4)	0.32 ± 0.26 (*N* = 6)	n.s
145	0.36 ± 0.17 (*N* = 4)	0.84 ± 0.80 (*N* = 6)	n.s
175	0.81 ± 0.24 (*N* = 4)	1.44 ± 1.32 (*N* = 6)	n.s
205	1.05 ± 0.25 (*N* = 4)	1.86 ± 1.54 (*N* = 6)	n.s
235	1.48 ± 0.84 (*N* = 4)	1.90 ± 1.22 (*N* = 6)	n.s

Measurement of pH values over a 235—minute period showed that the decline in arterial blood pH for the ventilated animals, receiving acidified ethanolic − dextrose solution + mannitol was very similar to that achieved by the ventilated rabbits given an acidified ethanolic—dextrose solution, decreasing to 7.22 ± 0.06 from a reference range of 7.46 ± 0.02 (*N* = 6), *p* < 0.05. Urinary pH increased to 6.02 ± 0.74 from a control of 5.83 ± 0.45 (*N* = 6), n. s. Net acid excretion (NAE) increased to 10.11 ± 2.16 from a baseline value of 3.92 ± 2.54 μmol min^−1^ (*N* = 6), *p* < 0.05.

**FIGURE 2:**
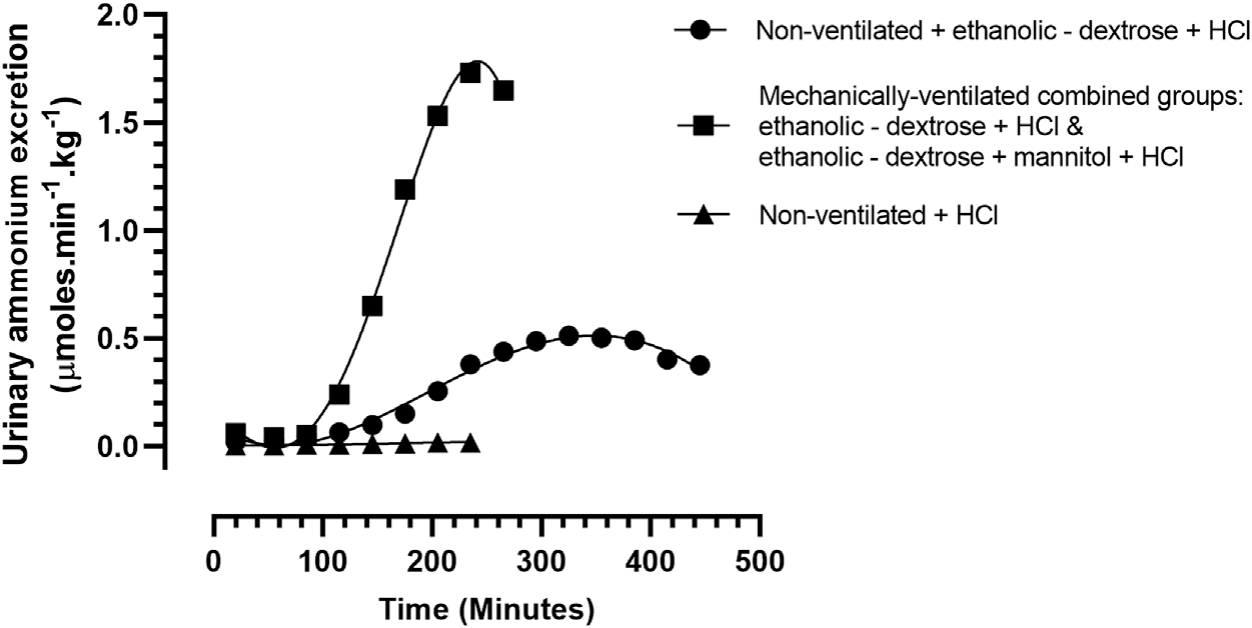
Urinary ammonium excretion rate, in rabbits treated according to Protocols 1–4: Protocol 1 (N = 9): 0.9% NaCl + 0.1 M HCL, non – mechanically ventilated. Protocol 2 (N = 5): Ethanolic - dextrose + 0.1 M HCl, non – mechanically ventilated (N = 5). Protocols 3 + 4 (N = 10): 4 rabbits administered ethanolic - dextrose + 0.1 M HCl and 6 rabbits administered ethanolic – dextrose + mannitol + 0.1 M HCl, both groups mechanically ventilated.

**FIGURE 3:**
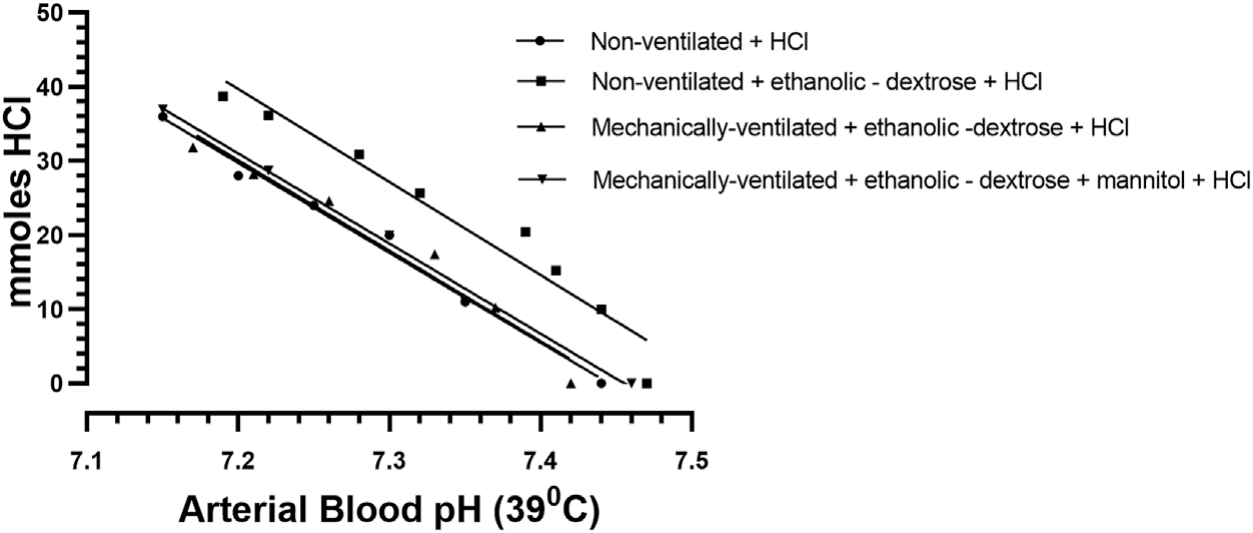
The titration of arterial blood at 39°C in each of four different rabbit subgroups, each having received a regimen, according to Protocols 1–4: Protocol 1 Control Group (N = 9): 0.9% NaCl + 0.1 M HCL, non – mechanically ventilated. Protocol 2: Ethanolic - dextrose + 0.1 M HCl, non – mechanically ventilated. Protocol 3: Ethanolic - dextrose + 0.1 M HCl, mechanically ventilated. Protocol 4: Ethanolic - dextrose + mannitol + 0.1 M HCl, mechanically ventilated.

The raw data for urinary ammonium output, obtained from each of the ten ventilated rabbits, treated according to protocols 3, 4, were pooled, and analysed statistically. The computed mean values were compared with similar data obtained for the two groups of non—ventilated rabbits, treated according to protocol 1 (0.9% NaCl +0.1 M HCl) and protocol 2 (acidified ethanolic—dextrose solution), as shown in Figure 2. The more rapid onset and superior magnitude of the ammoniagenic response, achieved by the mechanically—ventilated groups of acidotic rabbits (protocol 3: acidified ethanolic − dextrose solution and protocol 4: acidified ethanolic − dextrose solution + mannitol), is clearly visible. However, despite this substantial renal ammoniagenic response, the non—ventilated group of rabbits, receiving an acidified ethanolic dextrose solution, displayed the superior resilience to the ensuing acidosis, in comparison to the non—ventilated control group (0.9% NaCl +0.1 M HCl) and the two ventilated groups of animals (Figure 3). In quantitative terms, the intravenous infusion of 40mmoles of HCl titrated the arterial blood to pH 7.2 from control values in the non—ventilated group receiving an acidified ethanolic dextrose solution, whereas 30 mmoles HCl was required to bring the blood to the same pH endpoint, in the case of the control group of non—ventilated rabbits (0.9% NaCl +0.1 M HCl). No significant difference in acid resilience was observed between the control group of non—ventilated rabbits and the two groups of artificially ventilated animals, through—out the entire acidosis study.

The infusion of a solution, containing 5% dextrose, 3% ethanol, 5% mannitol, and 0.1 M HCl, produced a very significant increase in aldosterone excretion levels, which resulted in a correspondingly significant increase in the excretion of K^+^. The excretion of Na^+^ did not increase significantly, over the experimental period (Table 2).

**TABLE 2:** Urinary excretion of Aldosterone, K+, and Na+ during the intravenous infusion of a solution containing 5% dextrose, 3% ethanol, 5% mannitol, and 0.1 M HCl (Protocol 4).

Parameter	Control	235—Minutes	*p*-value
Aldosterone (pmoles.min-1)	3.0 ± 0.8 (*N* = 6)	7.2 ± 3.6 (*N* = 6)	<0.05
K^+^ (µmoles.min^−1^)	3.8 ± 1.5 (*N* = 6)	6.7 ± 1.5 (*N* = 6)	<0.05
Na^+^ (µmoles.min^−1^)	4.4 ± 4.3 (*N* = 6)	5.0 ± 4.1 (*N* = 6)	n.s

**FIGURE 4:**
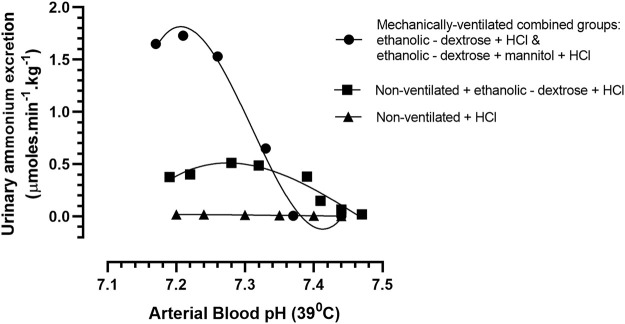
Arterial blood pH vs. urinary ammonium excretion rate in rabbits treated according to Protocols 1–4: Protocol 1 Control Group (N = 9): 0.9% NaCl + 0.1 M HCL, non - mechanically ventilated. Protocol 2 (N = 5): Ethanolic - dextrose + 0.1M HCl, non – mechanically ventilated (N = 5). Protocols 3 + 4 (N = 10): 4 rabbits administered ethanolic - dextrose + 0.1 M HCl and 6 rabbits administered ethanolic – dextrose + mannitol + 0.1 M HCl, both groups mechanically ventilated.

The relationship between arterial blood acidity and urinary ammonium excretion in the mechanically—ventilated groups of acidotic rabbits (acidified ethanolic − dextrose solution and acidified ethanolic dextrose + mannitol) was uniquely different to that which had been observed in the non-ventilated group of acidotic animals (non-ventilated + acidified ethanolic − dextrose solution). In the case of the two groups of rabbits that were mechanically ventilated with room air, urinary ammonium excretion increased rapidly, and in a linear fashion, in response to an increased arterial blood acidity, until an arterial blood pH of 7.21 was reached, after which the ammonium excretion rate began to slow down (Figure 4). In contrast, the non—mechanically ventilated animals displayed a poorer correlation between the increase in urinary ammonium excretion and the increase in arterial blood acidity over the course of the acidification process. Furthermore, the speed and magnitude of the ammoniagenic response was considerably less, and the maximum urinary excretion rate of ammonium occurred at the much higher arterial blood pH of 7.28, in comparison with a mean value of pH of 7.21, that was noted in the two mechanically ventilated groups of animals. These notable differences between the two groups of rabbits highlight the very significant role that the respiratory system plays in this species, in controlling arterial blood pH. Through the mechanism of hyperventilation, the non—mechanically ventilated rabbits, receiving the acidified ethanolic—dextrose solution, were able to reduce the arterial blood pCO_2_ in proportion to the declining buffering capacity of the plasma, which was the result of acid being intravenously infused, and non-volatile acidic compounds being formed *in vivo*, due to the metabolism of ethanol. Even though the respiratory system was not incapacitated in the non—mechanically ventilated control group, receiving 0.9% NaCl +0.1 M HCl, their inability to generate comparable amounts of plasma bicarbonate and urinary ammonia, would have accounted for their reduced buffering capacity and lower acid resilience (Figure 4). There was no evidence of “dilution acidosis” in any of the nine control rabbits, who had been volume expanded with 0.9% saline, prior to being rendered acidotic *via* infusion of 0.1 M HCl.

## 6 Discussion

It is well known that herbivorous species such as the guinea pig and rabbit have a very low acid tolerance (Richterich et al., 1958). This study demonstrates that acid tolerance in the non—mechanically ventilated rabbit can be substantially enhanced when acidosis is induced by the intravenous infusion of a solution containing 5% dextrose, 3% ethanol and 0.1 M HCl (Leonard and Orloff, 1955). The metabolism of ethanol to acetate may provide the key to accounting for the increased acid resilience, that was observed by us.

The hepatic metabolism of ethanol to acetate in the rabbit involves three pathways. These include a pathway catalysed by alcohol dehydrogenase (ADH), of which there are several isoenzymes with different values for Km and Vmax, and two oxidative non—ADH pathways (Fujimiya et al., 1989). Pharmacokinetic studies of acetate during ethanol oxidation in the rabbit showed that the fraction of a dose of ethanol converted to acetate was 0.54, and that the elimination of acetate followed a Michaelis—Menten kinetic model, with Vmax & Km values of 40.8 ± 14.1 mM h^−1^ and 0.47 ± 0.19 mM, respectively (Fujimiya et al., 2000).

Like the kidneys of other species, the rabbit kidney has a high capacity to utilize acetate as a substrate (Chauvin et al., 1997). Following uptake by renal cells, acetate readily gains entry to mitochondria, and thereafter undergoes an intricate series of metabolic cycles during its metabolism, involving both the mitochondrion and cytosol (Chauvin et al., 1997; Dugelay et al., 1999; Conjard et al., 2002). The oxidative metabolism of acetate would have generated considerable amounts of bicarbonate, which would have been returned to the blood stream, directly bolstering plasma bicarbonate levels. Furthermore, through the stimulation of proximal tubule ammoniagenesis, the metabolism of acetate would have facilitated the provision of ammonia base, that would have enabled the α-intercalated cells in the collecting duct, increase the unloading of acid into the urine, while returning parallel amounts of additional bicarbonate to the blood stream. This hypothesis is supported by the observations of a French research group (Chauvin et al., 1997), who noted in ^13^C NMR studies of rabbit kidney tubules, that acetate induced stimulation of flux through glutamate dehydrogenase (GDH) in the oxidative deamination direction, increased α-ketoglutarate dehydrogenase, and citrate synthase catalysed reactions, whilst, at the same time, inhibiting flux through glutamine synthetase and the glutamate transamination pathways. As glutamate is a well-known end-product inhibitor of PDG, a reduction in intra-mitochondrial glutamate levels would also have favoured the activation of PDG, resulting in additional ammonia production by the proximal tubules. However, because of a relatively low renal PDG activity in the rabbit, the contribution of this pathway to ammoniagenesis would have been significantly less than *via* GDH.

Acid loading has been shown to induce renal carbonic anhydrase mRNA expression (Tsuruoka et al., 1998). Furthermore, the activity of carbonic anhydrase (CA) is known to be influenced by the arterial blood pCO_2_ status. For example, hypocapnia leads to a decrease renal H^+^ secretion, while hypercapnia increases H^+^ secretion by the kidneys. Evidence for this close inter-relationship was demonstrated in this study by the results showing that much higher urinary ammonium excretion rates were achieved in the mechanically ventilated rabbits in comparison to the non-ventilated animals, as both groups became acidotic. The intravenous infusion of the acidified ethanolic dextrose solution would have shifted the carbonic anhydrase catalysed reaction to the left, increasing the arterial blood pCO_2_ in both groups. While the non-ventilated animals would have been able to hyperventilate and exhale the excess carbon dioxide, mechanical ventilation would have incapacitated this homeostatic mechanism.

An elevating extracellular pCO_2_, due either to intravenous acid loading, or the direct accumulation of volatile acid through respiratory inhibition, would have triggered a cascade of events inside the α—intercalated cells. These would have included the activation of intracellular carbonic anhydrase by CO_2_, increasing the intracellular HCO_3_^−^concentration, which in turn would have activated soluble adenyl cyclase (sAC) to produce cAMP, culminating, through the activity of phosphokinase A (PKA), in the insertion of vacuolar proton pump (V-ATPase)—containing vesicles into the luminal cell membrane, facilitating increased H^+^ secretion. The unloading of H^+^ into the urine by the α-intercalated cells would have been facilitated by the coordinated activities of key ammonia carriers (Weiner and Verlander, 2017). On the basolateral side, Rhesus B and C glycoproteins (Rhbg) along with a Na^+^—K^+^ ATPase would have enabled the uptake of NH_4_^+^ from the medullary interstitium into the α-intercalated cells, while on the luminal membrane of these same cells, Rhcg would have facilitated the secretion of NH_3_ into the tubular fluid, in parallel with the secretion of H^+^.

The intravenous infusion of hypertonic solutions is known to induce volume expansion, resulting in hyponatraemia, and increased aldosterone secretion. Increasing plasma levels of aldosterone would have enhanced the electrogenic transport of Na^+^ *via* the epithelial sodium channel (ENaC), located on the apical membranes of the principal cells in the CD, increasing lumen—negative transepithelial voltage (Pearce et al., 2015). The increased negativity of the tubular lumen would have stimulated the renal outer medullary potassium channel (ROMK) mediated secretion of K^+,^ while the increased DCT fluid flow rate would have further increased K^+^ secretion through the activation of the “big”/“maxi”—K^+^ channels (BK) (Subramanya and Ellison, 2014). The outcome of K^+^ hyper-secretion would be increased loss of K^+^ in the urine, resulting in reduced plasma K^+^ levels. Both hypokalemia and elevated plasma aldosterone levels would have further potentiated the proton—secreting and NH_3_ transporter (Rhcg) activities of the α-intercalated cells. It is thought that vasopressin, through its luminal V1a receptor, may play a critical role in mediating the stimulatory action of aldosterone in α-intercalated cells, by facilitating the nucleocytoplasmic transport of its mineralocorticoid receptor (MR) (Izumi et al., 2011).

Thus, the metabolism of acetate, through directly boosting plasma HCO_3_^−^ levels, coupled with indirectly facilitating enhanced net acid excretion by enhancing ammoniagenesis and urinary ammonia excretion, would have contributed immensely to delaying the impact of the intravenous acid loading process on the rabbit’s blood acid-base status. These metabolic factors, coupled with the robust nature of the rabbit’s respiratory system, would have contributed enormously to the increased systemic acid tolerance that was particularly demonstrated by the non—ventilated group of rabbits, in this study.

The very high urinary ammonium excretion rates reported in this study of acidotic rabbits have not previously been observed by other investigators. As acetate appears to mediate substantial renal production of ammonia in acidosis, it would be most important, in a future study, to examine if acetate induces a hyperammonaemia. To quantify such potential toxicity would be most important to ensure the safe use of acetate as a parenterally administered buffer in human medicine.

In terms of the rabbit as an herbivore, this study has demonstrated that this species does have its own unique physiological machinery, by way of its high respiratory minute volume and urinary ammonia buffer generating capacity, to be able to deal with high organic acid loads, originating either from acetate or lactate, both of which are known to be readily metabolised to equimolar concentrations of bicarbonate. The dominant nature of the rabbit’s respiratory system appears to reduce the magnitude of its renal response to an acid load, and in so doing, may account, in part, for the relatively very low urinary ammonium excretion values normally found in this acidotic species.

## Data Availability

The original contributions presented in the study are included in the article/supplementary material, further inquiries can be directed to the corresponding author.

## References

[B1] Bank N. (1961). The effect of buffer loading upon ammonium excretion in the dog. J. Clin. Invest. 40, 573–578. 10.1172/JCI104285 13686595 PMC290754

[B2] Bergman E. N. (1990). Energy contributions of volatile fatty acids from the gastrointestinal tract in various species. Physiol. Rev. 70, 567–590. 10.1152/physrev.1990.70.2.567 2181501

[B3] Blaxter K. (1962). The energy metabolism of ruminants. Springfield, Ill 332pp: Charles C Thomas.

[B4] Chapot G. Barrault N. Müller M. Dargnat N. (1972). Comparative study of Pa CO2 in several homeothermic species. Am. J. Physiol. 223, 1354–1357. 10.1152/ajplegacy.1972.223.6.1354 4641628

[B5] Chauvin M. F. Megnin-Chanet F. Martin G. Mispelter J. Baverel G. (1997). The rabbit kidney tubule simultaneously degrades and synthesizes glutamate. A 13C NMR study. J. Biol. Chem. 272, 4705–4716. 10.1074/jbc.272.8.4705 9030522

[B6] Cheema-Dhadli S. Halperin M. L. (1979). Role of the mitochondrial anion transporters in the regulation of ammoniagenesis in renal cortex mitochondria of the rabbit and rat. Eur. J. Biochem. 99, 483–489. 10.1111/j.1432-1033.1979.tb13279.x 499211

[B7] Conjard A. Dugelay S. Chauvin M. F. Durozard D. Baverel G. Martin G. (2002). The anaplerotic substrate alanine stimulates acetate incorporation into glutamate and glutamine in rabbit kidney tubules. A (13)C NMR study. J. Biol. Chem. 277, 29444–29454. 10.1074/jbc.M111335200 12019262

[B8] Cunarro J. A. Weiner M. W. (1974). A comparison of methods for measuring urinary ammonium. Kidney Int. 5, 303–305. 10.1038/ki.1974.41 4850179

[B9] Dugelay S. Chauvin M. F. Megnin-Chanet F. Martin G. Laréal M. C. Lhoste J. M. (1999). Acetate stimulates flux through the tricarboxylic acid cycle in rabbit renal proximal tubules synthesizing glutamine from alanine: A 13C NMR study. Biochem. J. 342, 555–566. 10.1042/bj3420555 10477267 PMC1220497

[B10] Fujimiya T. Li Y. J. Ohbora Y. (2000). Michaelis-Menten elimination kinetics of acetate during ethanol oxidation. Alcohol. Clin. Exp. Res. 24, 16S–20S. 10.1111/j.1530-0277.2000.tb00005.x 10803773

[B11] Fujimiya T. Yamaoka K. Fukui Y. (1989). Parallel first-order and Michaelis-Menten elimination kinetics of ethanol. Respective role of alcohol dehydrogenase (ADH), non-ADH and first-order pathways. J. Pharmacol. Exp. Ther. 249, 311–317. 2709333

[B13] Guyton A. C. (1947). Measurement of the respiratory volumes of laboratory animals. Am. J. Physiol. 150, 70–77. 10.1152/ajplegacy.1947.150.1.70 20252828

[B14] Halperin M. L. Cheema-Dhadli S. (1980). Regulation of renal ammoniagenesis in the rabbit. Int. J. Biochem. 12, 135–137. 10.1016/0020-711x(80)90056-7 7399013

[B15] Izumi Y. Hori K. Nakayama Y. Kimura M. Hasuike Y. Nanami M. (2011). Aldosterone requires vasopressin V1a receptors on intercalated cells to mediate acid-base homeostasis. J. Am. Soc. Nephrol. 22, 673–680. 10.1681/ASN.2010050468 21415155 PMC3065223

[B16] Leonard E. Orloff J. (1955). Regulation of ammonia excretion in the rat. Am. J. Physiol. 182, 131–138. 10.1152/ajplegacy.1955.182.1.131 13248956

[B17] Martínez-Ródenas F. Oms L. M. Carulla X. Segura M. Sancho J. J. Piera C. (1989). Measurement of body water compartments after ligation of the common bile duct in the rabbit. Br. J. Surg. 76, 461–464. 10.1002/bjs.1800760512 2736357

[B18] Michoudet C. Chauvin M. F. Baverel G. (1994). Glutamine synthesis from glucose and ammonium chloride by Guinea-pig kidney tubules. Biochem. J. 297, 69–74. 10.1042/bj2970069 8280112 PMC1137791

[B19] Nowland M. H. Brammer D. W. Garcia A. Rush H. G. (2015). “Biology and diseases of rabbits,” in Laboratory animal medicine (Elsevier), 411–461.

[B20] Pearce D. Soundararajan R. Trimpert C. Kashlan O. B. Deen P. M. T. Kohan D. E. (2015). Collecting duct principal cell transport processes and their regulation. Clin. J. Am. Soc. Nephrol. 10, 135–146. 10.2215/CJN.05760513 24875192 PMC4284417

[B23] Richterich R. Goldstein L. Dearborn E. H. (1958). Ammonia excretion of the Guinea pig and rabbit. Am. J. Physiol. 192, 392–400. 10.1152/ajplegacy.1958.192.2.392 13508890

[B24] Richterich R. W. Goldstein L. (1958). Distribution of glutamine metabolizing enzymes and production of urinary ammonia in the mammalian kidney. Am. J. Physiol. 195, 316–320. 10.1152/ajplegacy.1958.195.2.316 13583168

[B25] Schroeder C. A. Smith L. J. (2011). Respiratory rates and arterial blood-gas tensions in healthy rabbits given buprenorphine, butorphanol, midazolam, or their combinations. J. Am. Assoc. Lab. Anim. Sci. 50, 205–211. 21439214 PMC3061421

[B26] Subramanya A. R. Ellison D. H. (2014). Distal convoluted tubule. Clin. J. Am. Soc. Nephrol. 9, 2147–2163. 10.2215/CJN.05920613 24855283 PMC4255408

[B27] Tsuruoka S. Kittelberger A. M. Schwartz G. J. (1998). Carbonic anhydrase II and IV mRNA in rabbit nephron segments: Stimulation during metabolic acidosis. Am. J. Physiol. 274, F259–F267. 10.1152/ajprenal.1998.274.2.F259 9486220

[B30] Walsh P. A. O'Donovan D. J. (2019a). An appraisal of the *in vivo* role of phosphate as a modulator of urinary ammonium and titratable acid excretion in the acidotic rabbit. J. Anim. Physiol. Anim. Nutr. 103, 1571–1577. 10.1111/jpn.13143 31241230

[B31] Walsh P. A. O'Donovan D. J. (2016). Collection of untainted urinary specimens from the bladder of an anesthetized rabbit. Lab. Anim. 45, 112–114. 10.1038/laban.953 26886657

[B32] Walsh P. A. O'Donovan D. J. (2020). The kinetics of inorganic phosphate excretion in the acidotic rabbit during intravenous phosphate loading: A pseudo-ruminant model. Sci. Rep. 10, 3988. 10.1038/s41598-020-61069-0 32132645 PMC7055221

[B33] Walsh P. A. O'Donovan D. J. (2019b). “The physiological conundrum of inorganic phosphate stimulation of urinary ammonium excfetion in the acidotic rabbit,” in Proceedings of the physiological society (London, UKScotland, U.K: Physiological SocietyAberdeen).

[B34] Weiner I. D. Mitch W. E. Sands J. M. (2015). Urea and ammonia metabolism and the control of renal nitrogen excretion. Clin. J. Am. Soc. Nephrol. 10, 1444–1458. 10.2215/CJN.10311013 25078422 PMC4527031

[B35] Weiner I. D. Verlander J. W. (2017). Ammonia transporters and their role in acid-base balance. Physiol. Rev. 97, 465–494. 10.1152/physrev.00011.2016 28151423 PMC5539407

[B36] Yu H. L. Giammarco R. Goldstein M. B. Stinebaugh D. J. Halperin M. L. (1976). Stimulation of ammonia production and excretion in the rabbit by inorganic phosphate. Study of control mechanisms. J. Clin. Invest. 58, 557–564. 10.1172/JCI108501 956385 PMC333213

